# Alpha-Synuclein Aggregation in Parkinson's Disease

**DOI:** 10.3389/fmed.2021.736978

**Published:** 2021-10-18

**Authors:** E. Srinivasan, G. Chandrasekhar, P. Chandrasekar, K. Anbarasu, A. S. Vickram, Rohini Karunakaran, R. Rajasekaran, P. S. Srikumar

**Affiliations:** ^1^Bioinformatics Lab, Department of Biotechnology, School of Bio Sciences and Technology, Vellore Institute of Technology (Deemed to be University), Vellore, India; ^2^Department of Bioinformatics, Saveetha School of Engineering, Saveetha Institute of Medical and Technical Sciences (SIMATS), Chennai, India; ^3^Department of Biotechnology, Saveetha School of Engineering, Saveetha Institute of Medical and Technical Sciences (SIMATS), Chennai, India; ^4^Unit of Biochemistry, Faculty of Medicine, AIMST University, Bedong, Malaysia; ^5^Unit of Psychiatry, Faculty of Medicine, AIMST University, Bedong, Malaysia

**Keywords:** Parkinson's, genes, alpha-synuclein, mutation, protein aggregation

## Abstract

Parkinson's disease (PD), a neurodegenerative disorder characterized by distinct aging-independent loss of dopaminergic neurons in substantia nigra pars compacta (SNpc) region urging toward neuronal loss. Over the decade, various key findings from clinical perspective to molecular pathogenesis have aided in understanding the genetics with assorted genes related with PD. Subsequently, several pathways have been incriminated in the pathogenesis of PD, involving mitochondrial dysfunction, protein aggregation, and misfolding. On the other hand, the sporadic form of PD cases is found with no genetic linkage, which still remain an unanswered question? The exertion in ascertaining vulnerability factors in PD considering the genetic factors are to be further dissevered in the forthcoming decades with advancement in research studies. One of the major proponents behind the prognosis of PD is the pathogenic transmutation of aberrant alpha-synuclein protein into amyloid fibrillar structures, which actuates neurodegeneration. Alpha-synuclein, transcribed by SNCA gene is a neuroprotein found predominantly in brain. It is implicated in the modulation of synaptic vesicle transport and eventual release of neurotransmitters. Due to genetic mutations and other elusive factors, the alpha-synuclein misfolds into its amyloid form. Therefore, this review aims in briefing the molecular understanding of the alpha-synuclein associated with PD.

## Introduction

Parkinson's disease (PD) is the second most common neurodegenerative disorder that falls under the category of synucleinopathy (1). PD is characterized by distinct aging-independent loss of dopaminergic neurons in substantia nigra pars compacta (SNpc) region and the decrease in dopamine levels (2). Possibly, PD leads to the loss of terminal ends of striatum, which occurs before the neuronal loss in SNpc and; it seems to be more significant in disease pathogenesis (3). About 95% of PD cases are sporadic with no genetic linkage. Mostly, PD has its mean age of onset at 55 years with increased incidences with aging (1). PD is the most common disorder in a range of disorders classified as Parkinsonism characterized by dopamine deficiency and striatal damage.

In early stages of PD, the patients are pre-symptomatic with possible pathological changes of dorsal motor nucleus of vagus in medulla and the anterior olfactory nucleus of olfactory bulb followed by changes in locus ceruleus neurons of pons and dopaminergic neurons of substantia nigra. Hence, the smell and taste disturbances may be early clinical features of PD. During later stages, the pathology spreads to amygdala, basal forebrain, and medial temporal lobe structures. Neocortex is affected in final stages of the disease. Wherein, these stages are based on Braak's PD staging scheme (2, 4).

PD is characterized by motor symptoms such as bradykinesia, hypokinesia, akinesia, hypomimia, hypophonia, drooling, swallowing problems, micrographia, decreased stride length during walking, rigidity (stiffness of limbs), postural instability, and resting tremors (5). Some of the non-motor symptoms of PD include sleep disorders, depression, memory impairment, lack of initiative, delayed response, slowed cognition, passiveness, psychosis, and confusion (6–9). Pain is the most common non-motor symptom in PD patients and it may occur even before the motor symptoms. Hence, these symptoms may not share the same pathogenic pathways. Nociceptive dysfunction in the peripheral primary afferent nerves leading to abnormal sensory input and degeneration has been hypothesized as a possible reason for the early-stage pain symptoms and impaired response to pain stimuli (10).

Other parkinsonian disorders and related atypical parkinsonian disorders are caused by multiple system atrophy (MSA), tauopathies, or progressive supranuclear palsy (PSP) and cortico-basal degeneration (CBD) (7).

The most pathological hallmark of PD is Lewy bodies (LB). Lewy bodies are intraneuronal inclusions that contain immunoreactive alpha-synuclein aggregates which may also contain various neurofilament proteins as well as proteins involved in proteolysis such as ubiquitin. Predominantly, the cell death is caused by disruption of nuclear membrane integrity and release of alpha synuclein aggregation promoting nuclear factors like histones. Alpha synuclein may spread to other cells by direct or indirect means once aggregation starts. When compared with unaffected normal individuals, around 50–70% of neurons are lost in this region, at the time of death in patients with PD (11–13). Some studies suggest that LBs are the cell's defensive mechanism to prevent intracellular protein aggregate accumulation, while other studies suggest LBs to have a pathogenic role in PD. Therefore, LBs are an area of controversy in PD. LB formation may activate pathways for neuronal dysfunction and cell death (2, 4).

Mutations in the alpha-synuclein gene are responsible for some familial cases of PD with LB, whereas mutations in the Parkin gene cause a parkinsonian syndrome without LB in early-onset cases. Furthermore, Parkin proteins cause ubiquitination of the alpha-synuclein by interacting with synphilin-1 and thus promote the formation of LB (14–16). Mutations in genes coding the proteins involved in ubiquitin-proteasome system (UPS) and some deubiquitinating enzymes have been linked to PD. UPS removes unwanted proteins inside the cell and maintains many intracellular processes for cell viability (5).

This review offers an analytical assessment of the literature designating the possible role of the genes involved in causing PD. The information was collected from the molecular, cellular, and computational studies from various library databases and search engines. Hence, this article helps in providing a better understanding over the impact of the alpha synuclein and its mutations causing PD in terms of the molecular and neurological perspectives.

## Overview About the Amyloidosis in Neurodegenerative Disorders

The pathological commonality among predominant neurodegenerative disorders such as Alzheimer's disease (AD), Huntington's disease (HD), PD, prion disorders, and amyotrophic lateral sclerosis (ALS) are protein aggregation and the formation of inclusion bodies. The aggregates also called as amyloids, are composed of fibers and fibrils consisting aberrant misfolded proteins rich in beta-sheets. Amyloid deposition is directly associated with cellular deterioration and neuronal malfunction. It is also insinuated to effectuate endoplasmic reticulum, oxidative stress, mitochondrial, and proteasomal dysfunction, thus eventually causing neuronal waning. While mutations are culpable behind protein misfolding, sporadic causes attributed to other genetic and environmental variables. Increasing reports from various studies have elucidated the better comprehension of biochemical pathways associated with protein aggregation. Chief pathways suspected to cause protein aggregation include unfolded states/unfolding intermediates mediated physical aggregation, aggregation propelled chemical linkages, or protein self-association and aggregation mediated via chemical degradations (17–20).

## Disordered Proteins Involved in Pathogenesis of Parkinson'S

**Figure 1:**
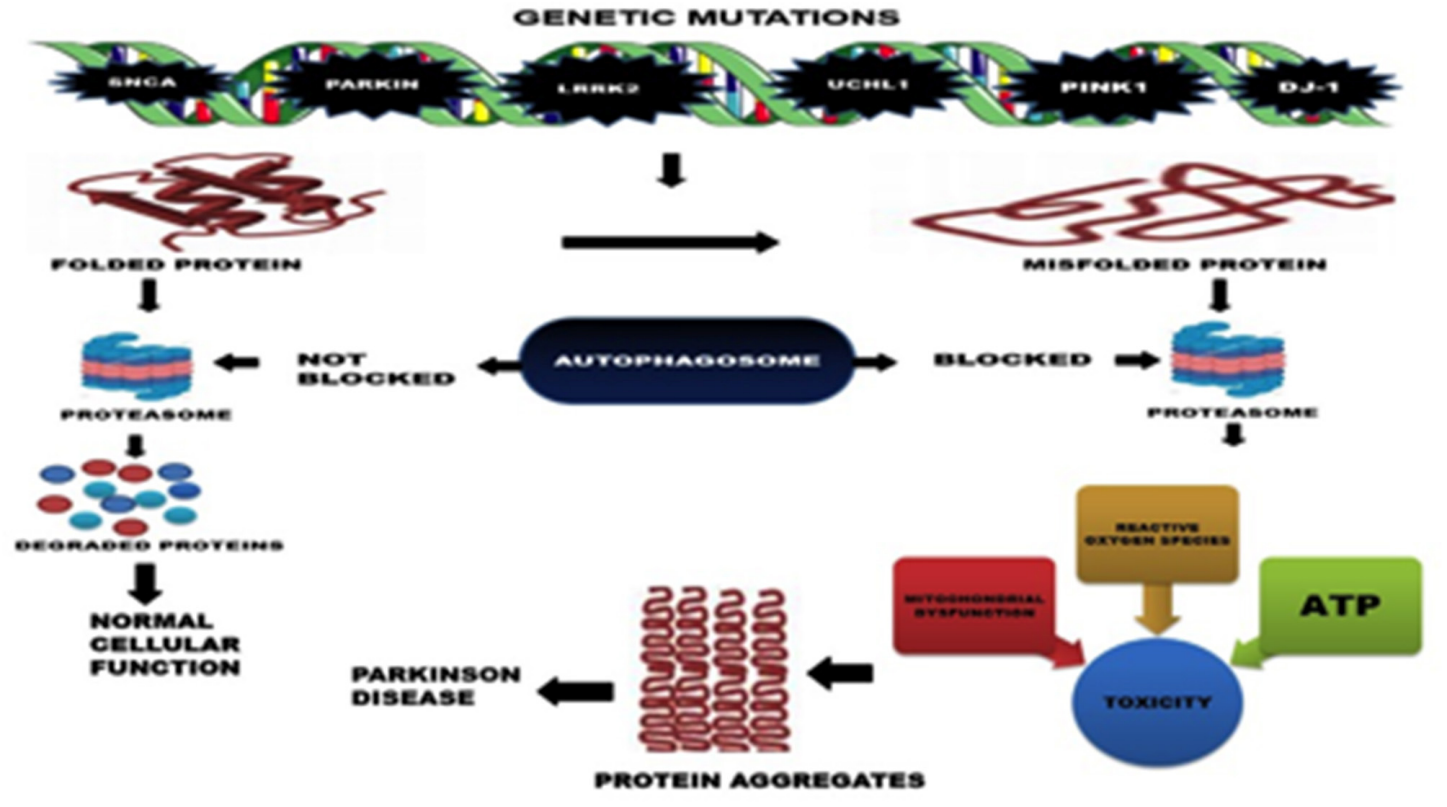
Mechanisms of Neurodegeneration in PD.

Primarily, the accumulation of misfolded proteins has been suggested to be the cause of PD. Mutations in alpha-synuclein (SNCA), ATP13A2, GBA, FBX07, VPS35, PLA2G6, DNAJC6, SYNJ1, UCHL1, parkin (PRKN), LRRK2, PINK1, and DJ-1 genes has been identified to cause familial early onset PD by causing abnormal protein conformations and disrupting the ability of cellular machinery to clear the misfolded proteins (Figure 1) (6, 21). Pdr-1 (Parkin gene) and PINK1 mutants in *C. elegans* have been found to exhibit defective dopamine dependent behavior.

ATP13A2 encodes cation-transporting ATPase 13A2 that partakes in maintaining mitochondrial, lysosomal, and neuronal integrity. Its chief function involves transporting divalent transition metal cations. On the other hand, Glucosylceramidase beta encoded by GBA gene, is implicated in glucocerebroside's hydrolyzation. While, FBXO7 gene translates into F-box only protein 7 that is involved in mitophagy. VPS35 encodes Vacuolar protein sorting-associated protein 35, which participates in autophagy by transporting proteins across Golgi apparatus and vesicular structures. Next, gene PLA2G6 translates into an 85–88 kDA protein, which is calcium-independent phospholipase A2. It is also involved in regulating phospholipid remodeling and fatty acids' release from phospholipids. In addition, it has been implicated in prostaglandin and leukotriene production, and in nitric oxide/vasopressin mediated arachidonic acid release. DNAJC6, on the other hand, encodes putative tyrosine-protein phosphatase auxilin that partakes in neuronal clathrin-mediated endocytosis. Further, SYNJ1 translates into Synaptojanin-1, which is a phosphoinositide phosphatase enzyme that modulates PIP2 in membrane. Next, Ubiquitin carboxyl-terminal hydrolase isozyme L1 is encoded by geneUCHL1. It is a deubiquitinating enzyme that produces monomers of ubiquitin, suspected to regulate monoubiquitin's degradation in lysosomes. And, PRKN translates to E3 ubiquitin-protein ligase parkin, a ubiquitin ligase that participates in effacing damaged or misfolded proteins (22).

The pdr-1 and PINK1 mutants had greater accumulation of dysfunctional mitochondria with age, leading to the activation of mitochondrial ubiquitin proteasome response. By this way, the prevention of upregulation of ubiquitin protein response was found to reduce dopaminergic neuron lifespan and lead to their loss in *C.elegans* (23). PINK1 and Parkin were found to regulate the mitochondrial quality control in neurons by removing damaged mitochondria (with reduced membrane potential, increased ROS production, defective electron transport chain, or accumulation of unfolded proteins) through autophagy and thus replacing the damaged mitochondria. Mutations in either PINK1 or Parkin can alter this mechanism and lead to mitochondrial dysfunction, which is found to be prevalent in PD neurons (24). LRRK2 is the most common gene involved in sporadic PD, while the other gene defects cause only a small number of familial PD cases; however, they provide information on the proteins involved and disease mechanism (25). Point mutations in alpha-synuclein gene has been identified to cause early-onset PD in an autosomal-dominant way and the overexpression of gene has been found to cause late-onset or sporadic cases of PD (26). LRRK2 gene codes for the dardarin protein and this gene is the most common cause of familial or sporadic PD. LBs have been found in PD cases involving LRRK2 (27). LRRK2 G2019S mutation (substitution of glycine to serine at codon 2019) accounts for the majority of familial cases and 1.6% of sporadic cases of PD even though its prevalence is variable (28). Single gene mutations in Parkin and DJ-1 cause early-onset PD and these mutations are inherited by autosomal-recessive pattern. Abnormalities in mitochondrial Complex I of oxidative phosphorylation enzyme pathway have been consistently found to cause mitochondrial defects by inducing oxidative stress, increasing the production of reactive oxygen species along with superoxides that can target the electron transport chain to accelerate their own production and energy failure leading to PD pathogenesis (29). Dopaminergic neurons are particularly vulnerable to oxidative stress reactive oxygen species mediated mitochondrial dysfunction because the metabolism of dopamine produces superoxides radicals, hydrogen peroxide, and DA-quinone (produced by auto-oxidation of dopamine) which damage the cells. However, the directs links between ROS generation, defects in oxidative phosphorylation, and PD pathology are not strong and convincing, because of the rare occurrence of Parkinsonism in patients with mutations affecting oxidative phosphorylation. Mitochondrial defects have been suggested to cause cell death and dysfunction in PD, which could occur due to the inherited defective mitochondrial DNA or mutations in mitochondrial genome caused by possible systemic toxicity. Deregulation of kinase signaling, disruption of signaling mechanisms in dopaminergic neurons and endoplasmic reticulum stress are also key molecular mechanisms in PD (30, 31). PINK1 gene codes for a mitochondrial complex that has been shown to be responsible for autosomal-recessive form of PD, but it is not a major risk factor of sporadic PD. Programmed cell death has been suggested to cause the neuron cell death in PD, but whether it causes cell death due to abnormal pathway or to clear cells injured and damaged by the pathological mechanisms of PD is still not clear (6, 8, 32).

Furthermore, Franco et al. have compiled the aforementioned genes' functionalities in terms of their biochemical, biomolecular, network interactions, and pathway analysis from various *in silico, in vitro*, and *in vivo* studies, to formulate putative mechanism: due to genetic mutations observed in the afore mentioned list of genes, involved in protein processing and vesicular trafficking, native alpha synuclein's normal processing is hindered and altered (22).

## Structural Architecture and Disease-Causing Mutations in Alpha Synuclein (AS)

Alpha synuclein is a small (14 kDa), intrinsically disordered protein with 140 amino acids that is highly charged and coded by *SNCA* (synuclein) gene, which is mainly expressed in CNS (33). Alpha synuclein is predominantly found at the presynaptic terminals, where it associates with synaptic vesicles. Alpha synuclein belongs to the synuclein family, which also includes gamma and beta synucleins. One percentage of the total proteins of neuronal cytosol are comprised of AS. AS has an amphipathic N-terminus consisting of 7 imperfect sequence repeats of 11 residues with a possible alpha helix structure that facilitates lipid binding and a potential role in aggregation. Further, the non-amyloid component (NAC) at the C- terminus facilitates calcium binding and inhibits protein aggregation (34).

Moreover, NMR structure of human alpha-synuclein reported by Ulmer et al. indicates that amino acids in-between 3–37 and 45–97 forms alpha-helices (curved), joined by linker (extended) organized in an unprecedented anti-parallel manner. Then, a notably high mobile tail spans in-between 98 and 140 amino acid range. The well-organized orientation of helical connector implicates a demarcated association with lipidic surfaces, indicating a notion that, when adhered to synaptic vesicles with larger diameter, it can function as a modulator between the aforementioned structure and an uninterrupted helical model that was postulated earlier (35).

AS is involved in synucleinopathies like PD (both familial and sporadic), multiple systems atrophy and dementia with LB; however, the physiological function of AS is still unknown. Researchers suggest that AS could potentially play a role in cell function regulation, dopamine release regulation, vesicular trafficking, and oxidative stress. Removal of AS gene in mice has been found to cause the loss of dopaminergic neurons, striatal dopamine reduction, and absence of dopamine-induce locomotive responses mediated by dopamine transporter (DAT) (4). Missense point mutations of the N-terminal (A53E, A53T, A30P, E46K, H50Q, and G51D) have been strongly correlated with autosomal dominant form of PD, while, the duplication and triplication of AS gene have been shown to be involved in familial PD cases with early onset (36). Mutant AS proteins vary in only a few amino acid residues but result in a significant change in their conformation and the type of aggregates formed (37). There is no explanation for this phenomenon to date. AS inclusions are found to be usually hyperphosphorylated at various sites including Ser129, Ser87, and Tyr125 in LB (38, 39). Mutant AS protein (A30P and A53T disease mutations) involved in familial PD have been shown to be structurally defective for membrane binding, leading to alteration of the protein's binding properties (40). Wild-type AS has been observed to form two different dimers and; the single point mutations (A30P, E46K, and A53T) have been suggested to promote dimerization of AS. Moreover, the structural homogeneity of these dimers has been suggested to lead in different aggregation pathways (41). In a mutation-frequency analysis conducted on Japanese patients, the SNCA p.A53V homozygous mutation was found to cause distinct phenotype of progressive Parkinsonianism and cognitive decline similar to SNCA missense mutation. Particularly, the two newly discovered mutants of AS viz., A18T and A29S were found to aggregate faster than wild-type AS with greater propensity for aggregation. Furthermore, the A18T mutant was found to have faster aggregation kinetics compared to A29S and hence, it makes the protein more sensitive to aggregation by modifying its native conformation (42). Of note, the broken helix structure of AS, which consists of two antiparallel membrane bound helices connected by a non-helical linker causes the protein to interact with synaptic vesicles docked at the plasma membrane. Phosphorylation of tyrosine at position 39 (Y39 phosphorylation) in AS, *in vitro*, was found to free the protein from the membrane surface of vesicles by decreasing the binding of helix-2 in the broken helix state. In addition, this effect was found to be similar to the effect of G51D mutation (43). The peptide (1a) consisting of residues 36–55 of AS was found to form a beta hairpin structure that subsequently assembled into a triangular trimer. Full length AS has been suggested to from such an assembly with evidences from molecular modeling. Also, this 1a peptide was able to bind anionic lipid bilayers membranes and nucleate the oligomerization of AS (44). Recently in a study of 426 Italian PD patient, the 263 bp allelic variant of Rep-1 (D4S3481 microsatellite), present upstream of the SNCA gene translation start site, was found to raise the risk of hallucinations and dementia in patients carrying this variant compared to the non-carriers (45). Chinese PD patients have been found to have lower resting-state brain activity in the lingual gyrus and left caudate, when the amplitude of low-frequency fluctuation (ALFF) values of the brain was compared between the PD patients and healthy controls. Furthermore, the participants carrying the rs894278 single nucleotide polymorphism in SNCA gene, also called as the G allele, were found to have lower ALFF values in the right fusiform compared to non-carriers of the G allele. This study suggests that PD may also alter the brain connections (46).

Apart from aforementioned mutations, certain genetic polymorphism in SCNA can be accredited toward enfeebling sporadic PD. Reports have shown that SNPs: rs7684318, rs894278, and rs2572324 have be known to enhance susceptibly toward sporadic PD. Further, missense mutations A29S and A18T were found in patients with sporadic PD (47). Also, a study conducted in Japanese population states that SNPs rs2736990 and rs356220 were considerably associated with sporadic PD risk (48).

Due to various genetic mutations and other obscure biomolecular circumstances, AS pathogenically misfolds and transmutes into amyloid fibrils that are rich in beta-sheets. Solid state NMR structure of pathogenic AS fibrils was presented by Tuttle et al. it was reported that AS fibril had more than 200 distinctive long-range distance restraints that delineates a consensus structure possessing characteristics such as hydrophobic-core residues and in-register β-sheets, and diverse residual framework such as a glutamine ladder, intermolecular salt bridge, small residues mediated close backbone interactions, and various other steric zippers that stabilizes the orthogonal Greek-key topology (49). However, it should be noted that amyloid fibrils are usually polymorphic: capable of existing in multiple viable forms (50). Recently, Guerrero-Ferreira et al. had reported two new polymorphic structures of AS fibrils, which evince distinct morphological features (51).

## Neurotoxic Effect of as in PD

AS is associated with calcium homeostasis and its overexpression may disrupt calcium homeostasis making dopaminergic neurons vulnerable to damage (52). Besides, AS has been suggested to have good affinity to phospholipid membranes, especially the synaptic vesicles with a preference for membranes with high curvature and specific membrane microdomains (53). AS has been found to regulate synaptic vesicle distribution and presynaptic terminal size. In specific, the N-terminal alpha helix helps in its lipid-binding capability (54). Increase in AS levels has been suggested to decrease dopamine as well as glutamine neurotransmission by interacting with the SNARE protein complex, modulating endoplasmic reticulum-golgi vesicular trafficking, inhibiting vesicular priming, and reducing synaptic contact (38). Overexpressed monomeric wild-type AS has been shown to inhibit vesicle endocytosis and impair neurotransmission. AS has been suggested to cause PD by disrupting the synthesis, storage, recycling, reuptake, and efflux of dopamine (55). Increased levels of AS has also been found to reduce active tyrosine hydroxylase (TH) enzyme that is involved in the production of dopamine by stabilizing TH in its inactive form. AS overexpression was found to attenuate vesicular monoamine transporter 2 (VMAT2) activity. Thus, the dopamine is stored in synaptic vesicles via VMAT2 after its production to reduce the oxidative damage from its metabolites. Increased cytosol concentration of dopamine due to the reduction of VMAT2 activity by AS has been proposed as a possible neurotoxic pathway in PD (56). Dopamine transporter (DAT) has been associated with dopamine trafficking, but whether it increases or decreases DAT levels is still a debate because of evidence supporting both sides (57). DAT knock-out mice showed high extracellular dopamine levels and low intraneuronal dopamine concentration; thus, DAT is important for neurotransmission and its activity (especially decreased dopamine uptake as well as increased dopamine clearance and efflux) upon disruption by AS can cause PD. Most of these proposed functions of AS rely on its membrane binding capacity (38, 39, 58–61). *In vivo*, AS is distributed as unstructured, cytosolic, soluble, and partially membrane-bound state. Of note, the equilibrium between the structured and unstructured states or the balance of the order and disorder of AS conformations at the surface of membranes has been suggested to influence the biological functions of the protein and also lead to the aggregation of the protein due to detachment from the membrane (62). Membrane binding by AS requires a conformational transition into a highly helical state and this is promoted by the amphipathic N-terminal. Moreover, the C-terminal of AS was found to be highly disordered and the NAC domain has been found to be highly structured in beta-sheet conformation in the amyloid state of AS (59). AS oligomers were found to stabilize and enhance pre-existing defects in supported lipid bilayers (SLBs) of 1-palmitoyl-2-oleoyl-sn-glycero-3phosphocholine/1-palmitoyl-2-oleoyl-sn-glycero-3-phospho-L-serine(POPC/POPS). Particularly, the exposed lipid acyl chains at the edges of membrane defects were suggested to promote the membrane-oligomer interactions resulting in the development of fractal domains lacking lipids. The growth of membrane damage pattern did not depend on the lipid- oligomer interaction suggesting an oligomer-dependent, diffusion limited extraction mechanism for the enhancement of membrane damage by AS oligomers (63).

**Figure 2:**
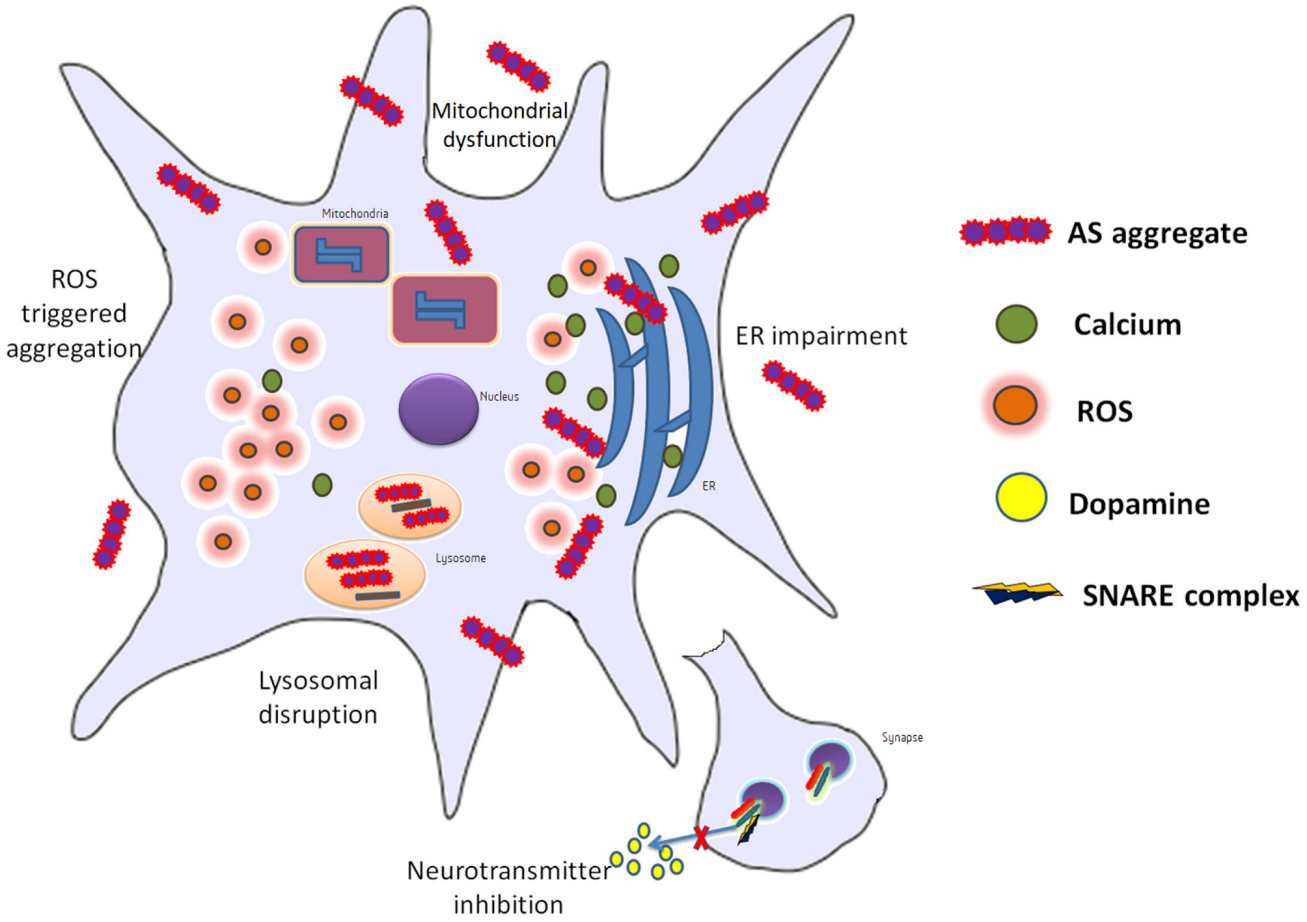
Info graph elucidating the various repercussions that can be observed in a neuron during PD.

AS may not form pores in membranes or induce damage by itself but enlarges previous membrane defects instead. The synphilin-1 proteins have been found to be accumulated abnormally in AS inclusions of synucleopathies (64). AS and neuroinflammation mediated by the inflammatory response through microglial activation have been suggested to potentiate each other. Misfolded AS may activate microglia by increased expression of TNF-α, IL-1β, IL-6, iNOS, and COX-2. Following stimulation by AS, receptors (TLRs, CD36, and FCγR) as well as signaling molecules (galectin-3, MMPs, and PHOX) have been proposed to join the microglial response to activate signaling pathways (NF-κB and MAPKs). These pathways further contribute to PD progression (65) (Figure 2).

Aberrant AS is also suspected to affect protein degradation systems. It was reported that malformed AS, under certain circumstances can trigger proteasomal dysfunction *in vitro* and *in vivo*. Further, mutant AS could also affect the lysosomes by impairing CMA, which in turn could induce macroautophagy that has a notable influence on neuronal survival. Also, AS is reckoned to influence dynamics of cytoskeletal system. Studies have shown that AS could interact and impact actin polymerization to affect cellular trafficking; substantial deviations on actin were reported, between native and A30P AS mutant. These findings indicate that apart from aberrant AS's neurotoxic role, it can also venture into aforementioned pathological digressions to propel PD disorder (34).

## Mechanism of as Aggregation

AS aggregation was found to be triggered by Cu(II) in conditions relevant to PD. Two independent Cu(II) binding sites with varying affinities in the N-terminus of AS were identified and the complex formation between the high affinity site of AS as well as Cu(II) was found to be critical to the metal-mediated fibrillation process. However, this complex formation is also hypothesized to make the protein vulnerable to oxidative damage. Presence of a truncated C-terminal was found to accelerate the process due to reduction of the non-aggregation activity of the C-terminal (66). The formation of N-terminal acetylated AS- Cu(I) complex at the N-terminal site I has been found to stabilize local conformations with alpha-helical secondary structure and restricted motility. Besides, the formation of this complex with stabilized helically folded structure may occur *in vivo* and also, affect the membrane binding and aggregation capability of the N-terminal acetylated AS (67). AS proteins lacking residues 109–140 in the C-terminal were found to form amyloid fibrils with strongly twisted beta-sheets, an increased beta-sheet distance, higher solvent exposure compared to monomeric wild-type AS, and incompatibility with the wild-type monomeric AS (68). Acetylation of the N-terminus of naturally occurring AS was found to perturb its ability to bind Cu^2+^ and the presence of the H50Q missense mutation along with the N-terminal acetylation prevented copper binding (69). Over expression of AS in frozen-hydrated primary midbrain neurons was found to increase intracellular Mn levels with reduction in levels of Ca, Zn, K, P, and S. Cu/Mn and Fe/Mn levels were found to have a strong correlation with AS overexpression. Mn release from the cells was also found to be reduced (70). Clioquinol was found to reduce the interaction between iron and A53T mutant AS in transgenic mice with improved phenotype (71). These studies suggest the involvement of transition metals in AS aggregation mediated PD pathology and suggest the regulation of these metal levels as a potential therapeutic strategy.

Tubulin polymerization promoting protein (TPPP/p25) has been found to co-localize with AS and induce its aggregation (especially oligomers and protofilaments) by forming a complex even though they are produced by different cells—oligodendrocytes and neurons, respectively. TPPP/p25 has been shown to be present in LB. Both of these are Neomorphic Moonlighting Proteins. TPPP/p25 is also a chameleon protein with high conformational plasticity and it is involved in physiological functions such as regulation of microtubules. Hence, targeting it with therapeutic drugs is difficult. A study on the chameleon TPPP/p25 protein- AS complex using NMR, CD spectroscopy, ELISA, and PCR followed by computational study using charge-hydropathy plot (CH-plot) and cumulative-distribution function plot (CDF-plot) has proposed that the interface between the two proteins in the pathological complex is a potential target for therapeutic drugs (72, 73). TPPP/p25α has also been shown to promote release of AS through exophagy and impairment of autophagosome formation/trafficking and lysosomal dysfunction preventing the degradation of AS (74). Cross-seeding between wild-type, A30P and A53T AS variants that vary in only one or two amino acid residues but result in different fibril morphologies were studied. Wherein, the study suggested that similarity of conformations of the seeds and monomers is essential for seed elongation (37). Further, the morphology of wild-type AS fibrils has been suggested to be determined by competitive growth between different polymorphs during fibrillation and slow maturation or annealing process of fibrils (75). AS was found to bind strongly with lipid membranes of large unilamellar vesicles with anionic or zwitterionic headgroups *in vitro* and this binding is suggested to reduce the protein-protein interactions of AS that lead to fibrillation. Recent updates in research pertaining to PD and AS is delineated in Table 1.

**Table 1:** Table delineating the recent research updates pertaining to AS and PD.

**S.no**	**Year of publishing**	**Author(s)**	**Title**	**Key research findings**	**References**
1	2020	Mellier et al.	The process of Lewy body formation, rather than simply α-synuclein fibrillization, is one of the major drivers of neurodegeneration	• Proposed seeding based model for AS fibrillation. • Elucidated the interaction between AS aggregates and cellular organelles like mitochondria ER etc.	(76)
2	2020	Ray et al.	α-Synuclein aggregation nucleates through liquid-liquid phase separation	Provides detailed characterization of AS's phase-separation behavior and its pathogenic transformation into aggregates.	(77)
3	2020	Perren et al.	The structural differences between patient-derived α-synuclein strains dictate characteristics of Parkinson's disease, multiple system atrophy, and dementia with Lewy bodies	Characterizes structural variations of pathogenic AD in various synucleinopathies.	(78)
4	2020	Stephens et al.	Extent of N-terminus exposure of monomeric alpha-synuclein determines its aggregation propensity	Identifies structural orientations and environmental conditions that furthers monomeric AS's aggregation.	(79)
5	2020	Zhao et al.	Parkinson's disease-related phosphorylation at Tyr39 rearranges α-synuclein amyloid fibril structure revealed by cryo-EM	Identifies PD's pathological outcome that reorients AS's 3D structure.	(80)
6	2020	Arlehamn et al.	α-Synuclein-specific T cell reactivity is associated with preclinical and early Parkinson's disease	Examines the association between α-syn-specific T cell responses and PD.	(81)
7	2020	Niu et al.	A longitudinal study on α-synuclein in plasma neuronal exosomes as a biomarker for Parkinson's disease development and progression	Findings indicate that AS found in plasma neuronal exosomes could server as biomarker in early PD detection and also as prognostic marker for the PD's progression.	(82)
8	2019	Bhattacharjee et al.	Mass Spectrometric Analysis of Lewy Body-Enriched α-Synuclein in Parkinson's Disease	Identified 20 AS variant species in PD patients.	(83)
9	2019	Levine et al.	α-Synuclein O-GlcNAcylation alters aggregation and toxicity, revealing certain residues as potential inhibitors of Parkinson's disease	Advocates O-GlcNAcylation as a potential therapeutic tool for regulating PD.	(84)
10	2019	Tian et al.	Erythrocytic α-Synuclein as a potential biomarker for Parkinson's disease	Finding indicate that AS levels are altered in PD patients' erythrocytesand peripheral erythrocytic could be a potential biomarker.	(85)
11	2019	Elfarrash et al.	Organotypic slice culture model demonstrates inter-neuronal spreading of alpha-synuclein aggregates	Characterizes how AS aggregates are formed and spreads across neurons.	(86)
12	2019	Landeck et al.	Toxic effects of human and rodent variants of alpha-synuclein *in vivo*	Quantifies the neurodegenerative effects of rodent and human AS variants.	(87)
13	2019	Brys et al.	Randomized phase I clinical trial of anti-α-synuclein antibody BIIB054	Delineates the phase I clinical trial outcomes of BIIB054 antibody against AS, which indicates that BIIB054 pose tolerability, safety, and pharmacokinetic propensities in PD patients.	(88)
14	2019	Kang et al.	Comparative study of cerebrospinal fluid α-synuclein seeding aggregation assays for diagnosis of Parkinson's disease	Formulates high accuracy AS seeding aggregation assay that can be used for PD diagnosis.	(89)
15	2018	Pihlstrom et al.	A comprehensive analysis of SNCA-related genetic risk in sporadic Parkinson's disease	Delineates AS encoding SCNA gene polymorphisms that are associated with sporadic PD.	(90)
16	2018	Longhena et al.	Synapsin III is a key component of α-synuclein fibrils in Lewy bodies of PD brains	Identifies Synapsin III as an integral part of AS fibrils in PD patients' brain.	(91)
17	2018	Faustini et al.	Synapsin III deficiency hampers α-synuclein aggregation, striatal synaptic damage, and nigral cell loss in an AAV-based mouse model of Parkinson's disease	Constitutes SynapsinIII as a crucial factor for AS aggregation and could be a potential target in mitigating PD.	(92)
18	2018	Papagiannakis et al.	Alpha-synuclein dimerization in erythrocytes of patients with genetic and non-genetic forms of Parkinson's Disease	Increased AS dimers were observed in PD patients, which could provide insights into PD prognosis and can be used as biomarker.	(93)
19	2018	Jankovic et al.	Safety and Tolerability of Multiple Ascending Doses of PRX002/RG7935, an Anti-α-Synuclein Monoclonal Antibody, in Patients With Parkinson's Disease: A Randomized Clinical Trial	Multiple doses of PRX002 antibodies was found to be safe and tolerable even resulted in robust interaction with peripheral AS.	(94)
20	2018	Zhang et al.	A Comprehensive Analysis of the Association Between SNCA Polymorphisms and the Risk of Parkinson's Disease	Delineates SNPs in SCNA gene that pose risk of developing PD rs11931074 and rs2736990 increased the risk in East Asian group rs181489, rs356219, rs356165, rs2737029, and rs11931074 increased the risk for European group.	(95)

## Overview of Therapeutics Targeting As

Presently, there are no effective therapeutics contrived for PD, but medication/surgery alleviates the symptoms and ameliorates motor impairments. A prudent way to effectively alleviate PD would be to target one of its crucial causatives, AS. Recently, various therapeutic strategies have been formulated, to encumber AS's toxic effect. One such strategy would be to control transmission by blocking AS receptors. LAG3-directed antibodies were reported to substantially regulate aberrant AS induced toxicity. Concurrently, silencing AS expression in mouse and rat brain models through shRNA and siRNA was also reported. Further, an oligomer regulator: Anle138b [3-(1,3-benzodioxol-5-yl)-5-(3-bromophenyl)-1H-pyrazole] was able to hinder the synthesis and accumulation of AS oligomers. Additionally, numerous small molecule-based inhibitors have been elucidated to impede AS aggregation. One such inhibitor: Methylthioninium was found to effectively control AS fibrillar inclusions *in vitro* and *in vivo*. Besides, numerous phytochemical and plant extracts were also found to effectively modulate AS aggregation in PD models. Lately, the use of phytochemicals to target AS aggregated has gained a lot of attention and focus in the research community (96, 97).

## Computational Studies On As

Using Monte Carlo (MC) computational methods (Replica Exchange MC and Canonical Protein MC, Molecular Dynamics (MD) based on CHARMM force field and AMBER force field were used in various studies to disintegrate the mutational effect on AS protein. On the other hand, virtual screening, docking using AUTODOCK (using Lamarckian Genetic Algorithm) and Interaction Potentials for assessment of protein druggability (MD- based DruGUI) were used to analyze the effect of drug molecules and other lead compounds on compact structures of AS in aggregates. Moreover, the regions of the protein that exhibit extended variable beta-sheet structure with a potential role in aggregation and the protein druggability for potential drugs inhibiting aggregation has been studied extensively (98). Notably, 332 genes that affect AS toxicity were identified using genome-wide screens of yeast. Later, these genes were used to map the human counterparts by using a newly developed computational method topology called Transpose Net that integrates Steiner prize-collecting approach with homology assignment through sequence, structure and interaction. Gene interaction profiles of ATP13A2/PARK9 and VPS35/PARK17 and network relationships between the genes LRRK2/PARK8, ATXN2, RAB7L1/PARK16, and EIF4G1/PARK18 were identified and confirmed in induced pluripotent stem cell (iPSC) derived neurons following the computational mapping (99).

Computational study using molecular dynamics simulations and modeling has been used to reveal varying aggregation effects in distinct protein conformational disorders (100–105). Thus, MD studies have revealed that the NAC domain of AS monomers strong interacts with the amyloid beta monomers. In specific, the cross-seeded NAC-amyloid beta oligomers were found to show polymorphism with the NAC oligomers preferring to form double layer conformations with amyloid beta over single layer conformations. Further, the self-assembled NAC oligomers with three beta strands connected by two turns were found to affect the secondary structure of self-assembled amyloid beta oligomers. Distinctly, the inner core distance values of NAC oligomers remained unchanged in cross-seeded NAC-amyloid beta oligomers but the inner core distance values in single layer amyloid beta conformations were decreased to form a compact and stable cross beta structure by strong hydrophobic interactions with the inner core domain. N-terminal of AS has been found to play an important role in the self-assembly of AS (1–140) into fibrils of cross-beta structure using models constructed by G-key 3D structure of self-assembled AS, NMR fibril structures, molecular dynamics simulations and GBMV calculations followed by analysis.

Recently, AS has been found to move into the nucleus by binding to Retinoic acid in a calreticulin dependent manner leading to increase toxicity and the AS in nucleus has been suggested to particularly increase the expression of PD-associated genes (ATP13A2, PINK1) in SH-SY5Y cells (106). N-terminal mutants of AS (D2A, D2P) were found to alter the N-terminal acetylation, protein level, stability, risk of neuron death and toxicity of AS in neuron as well as HEK293 models, showcasing the importance of the N-terminal amino acids and N-terminal acetylation in AS stability, toxicity, and aggregation (36). Overexpression of mutant A53T AS in HEK293 cells and rat dopaminergic neurons, was found to inhibit the 26S ubiquitin-proteasomal system and accumulation of AS phosphorylated at Ser129 was observed when the ubiquitin proteasomal system was inhibited (107).

## Interplay of Proteins and Genes in PD Pathogenesis

**Figure 3:**
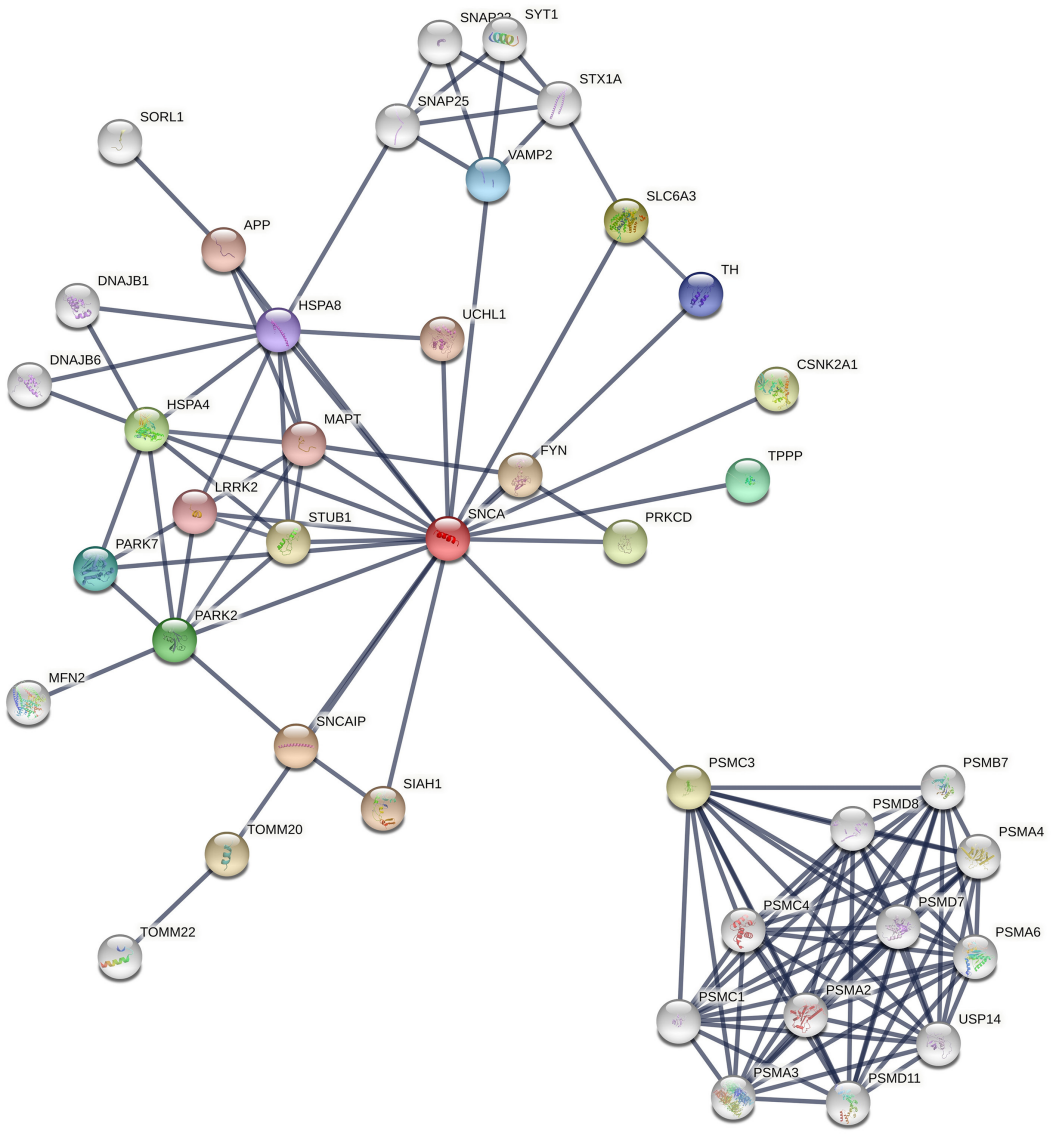
Snapshot of interaction network of the different Proteins involved in PD pathogenesis from STRING Database. The thickness of lines indicates the confidence score for the interaction between two proteins.

Synaptic vesicle endocytosis, mitochondrial dynamics, and Lysosomal/Proteasomal activity are three critical processes that become dysfunctional during PD pathogenesis. The dysfunction of these processes could be because neurons are vulnerable to oxidative stress as well as accumulation of damaged organelles or misfolded proteins. The over expression of mutant proteins overloads the defense mechanisms of the dopaminergic neurons leading to disease pathogenesis. PINK1 mediates mitophagy either independently or through the recruitment of Parkin to the damaged/depolarized mitochondrial membrane. The PINK1/Parkin pathway is also important for mitochondria fusion and fission dynamics. Hence, mutations/deficiency of these proteins can lead to accumulation of defective mitochondria in the cell and lead to oxidative stress. Oxidized proteins are transported from the mitochondria to lysosomes through mitochondrial vesicles (MDVs) during oxidative stress due to reactive oxygen species and this process has also been suggested to be affected by dysfunction of the PINK-1/Parkin pathway (Figure 3). Similarly, DJ-1 acts as a sensor of oxidative stress in the neurons and it has been suggested to move into damaged mitochondria. DJ-1 also protects cells from apoptosis due to stress. Mutations leading to loss of function of DJ-1 have been suggested to lead to reduced lysosomal activity and autophagy. Furthermore, increased expression of AS due to mutation, gene duplication or triplication has been suggested to cause mitochondrial fragmentation and oxidative stress. Glucocerebrosidase coded by GBA has also been suggested to degrade AS in lysosomes and reduce AS toxicity. It is also responsible for the conversion of glucosylceramide into membrane constituents. Mutations of GBA have been shown to lead to dysfunction of lysosomal autophagy, dysfunctional ubiquitin proteasome system, mitochondrial fragmentation, oxidative stress, and lead to defective lipid metabolism. Similarly, mutant LRRK2 under oxidative stress have been shown to cause apoptosis, defective vesicular trafficking and synthesis of defective/misfolded proteins (108, 109). Recently, mutated PINK1 expression in patient-derived neurons was found to lead to over-expression of LRRK2 at the mRNA and protein level (110). This suggests that PINK1 modulates LRRK2 level in neurons. DJ-1 has been suggested to regulate the level of PINK-1 and AS in neurons (111).

Parkin has been suggested to regulate the endolysosomal system by ubiquitination of Rab7. It has also been shown to interact with endophilin A (which is involved in autophagy and endocytosis) as well as other proteins involved in neuron survival. LRRK2 is also involved in endophilin A phosphorylation. Mutant AS has been suggested to prevent their own degradation in lysosomes which could be through Rab1a inhibition. Reduced activity of GCase1 due to GBA mutations has been shown to lead to accumulation of glucosylceramides and glycosphingolipids which may lead to greater production of Lewy Bodies and accelerate AS aggregation. Mutations in GBA are also suggested to cause the GCase protein to be stuck in the endoplasmic reticulum leading to ER stress, AS accumulation and interference with lysosomes. Misfolded AS aggregate and influence neurotransmission, synaptic vesicle exocytosis, recycling as well as endocytosis in the substantia nigra region. Furthermore, Aggregated AS has been suggested to target the retromer pathway of endolysosomal trafficking. The AS fibrils/filaments have been suggested to spread to surrounding regions through synapses in a prion-like manner through endocytosis and this has been suggested to evade lysosomal/proteasomal degradation through some way. Moreover, mutant/pathogenic LRRK2 with increased kinase-activity has been speculated to lead to exocytosis of misfolded AS fibrils along with increased endocytosis of such fibrils, which may help these fibrils to spread from cell-to-cell. AS has been suggested to promote the movement of pro-inflammatory monocytes into the substantia nigra region and also lead to the presentation of MHCII leading to inflammation and degeneration of neurons in this region. Although the activity of PINK-1/Parkin can help alleviate this process, mutated PINK-1/Parkin could not function this way (111, 112).

## Role of Personalized Medicine in Mitigating PD Pathology

Till now, PD's heterogenic pathology has been well-delineated. Further, it paves way for the formulation of personalized therapeutics, wherein it is surmised that pathophysiology and genetic might be crucial. Due to this heterogeneity, an imperative need for developing individualized pathology mitigating therapies have been imposed upon the medical society. Recent medical advancement has made us witness, treatments effectuated upon PD patients with specific genetic aberration.

Glucocerebrosidase is a common risk factor associated with PD. Mutations in GBA gene, which encodes glucocerebrosidase, are found predominantly among people from Netherlands and Ashkenazi Jews. Though GBA's role in PD remains obscure, targeted therapeutics can be steered toward restoration of enzyme function and regulation of glycosphingolipid turnover. Accordingly, gene therapy though adeno-associated virus expression glucocerebrosidase has shown positive findings in GBA pre-clinical studies. And reduced levels of alpha-synuclein aggregates were also reported in the same. Further, Ambroxol is currently being investigated in a PD clinical trial.

Like GBA, genetic mutations in LRRK2 are also prevalent in certain ethnicities and also considered to be a credible risk factor for autosomal dominant PD. Enhancing LRRK2 kinase seems to have a beneficial effect. At present, various structurally distinct LRRK2 inhibitors formulated by Merck, Pfizer, Genentech and GSK are in the pipeline.

Of note, the personalized medicine trails are more laborious and complicated than the typical clinical trial and can face various issues including the need for specific genotype patients in large number. Nonetheless, with the SCNA gene and its polymorphisms being studied and characterized extensively, it holds a great potential in the field of personalized medicine, to effectively mitigate toxic AS aggregates (113–115).

## Conclusion

Though AS's precise neuronal role remains obscure, it still persists as one of the crucial causalities behind PD. Its aberrant conformational change (due to genetic mutations and other unknown pathophysiological circumstances) into toxic amyloid fibrils disrupts cellular homeostasis and effectuates neuronal deterioration. The present review covers a broad range of information on the disease-causing gene AS related to PD disorder. By contrast with the previous century the current era of medical ailments and research studies had witnessed astonishing progress in understanding PD, particularly with respects to the clinical studies, genetics, pathophysiology and molecular mechanism of PD. Though prodigious, the intricacy in understanding the disease pathology of PD is considered to be a promising approach toward the significant therapeutic targets. Besides, personalized medicine for the treatment of the PD considering the specificity of the defective genes will be the forthcoming approach over the next decades in the field of medical research world. Moreover, the directed delivery of therapies toward the central nervous system crossing the blood brain barrier will embolden credible hope that aid in improvising the new treatment for the devastating PD disorder.

## Author Contributions

ES involved in conceptualization and writing and editing the final manuscript. GC and PC involved in sub-section of computational studies on alpha-synuclein. KA, AV, and RK involved in english correction and edited the manuscript. RR and PS involved in the design the review, supervision of the research work, and edited the final manuscript. All authors contributed to the article and approved the submitted version.

## Conflict of Interest

The authors declare that the research was conducted in the absence of any commercial or financial relationships that could be construed as a potential conflict of interest.

## Publisher's Note

All claims expressed in this article are solely those of the authors and do not necessarily represent those of their affiliated organizations, or those of the publisher, the editors and the reviewers. Any product that may be evaluated in this article, or claim that may be made by its manufacturer, is not guaranteed or endorsed by the publisher.
